# Notes and News

**Published:** 1904-04-02

**Authors:** 


### Notes and News.

The Queen has sent a donation of £100 to the Sister Matron of King's College Hospital for the removal scheme to South London.

Dr. R. Fuller, Personal Physician to the late Duke of Cambridge, has received the Royal Victorian Order of the Fourth Class from the King.

At the annual meeting of the Medical Graduates' College and Polyclinic, Sir William Broadbent's resignation as President of the College was announced. Sir William Broadbent has acted as President since the establishment of the College. The number of names on the register is continuously increasing, and the scope of the work increasing in usefulness.

On the motion for the third reading of the Bill referring to the Physical Condition of Children in Elementary Schools, before Parliament, Sir John Gorst, Mr. Cooks, and Sir William Anson spoke very strongly, urging attention to the fact that badly nourished children were harmed by the enforced education for which the public were paying.

The new wing at the Victoria Hospital for Children is now completed. The new building is practically a new hospital and has cost £35,000. There are sixteen wards on four floors and an isolation ward, which is placed in a separate building. A large operation theatre, *x*-ray department and clinical laboratory have been provided, with other offices.

The old Royal Infirmary buildings at Edinburgh have been sold to the University for £15,000, who will probably establish laboratories and research rooms on the site. It appears that about 34 years ago the managers of the old Infirmary sold their buildings and site to the University for £20,704, but the sale was stopped as the price was regarded as too small, and it was bought on behalf of the hospital for £30,000 by friends of the hospital. The sum of £15,000, it is argued, is quite disproportionate to the value to-day.

Infant life insurance is now prohibited in Belgium.

Upwards of £6,000 has been expended on sanitary improvements at the House of Commons during the past three years.

Sir Michael Foster delivered the first Huxley Lecture before the University of Birmingham last week, taking as his subject "The Life and Influence of Thomas Henry Huxley."

The Chicago Board of Education has appointed twelve medical inspectors of schools in the city. The teachers in the schools are required to keep the scholars under observance and to report to the inspectors.

A recent "Analysis of Vital Statistics for 1903" shows the remarkably low death-rate in Hornsey of 7.9 per 1,000 of the population. The death-rate of London at 15.7 per 1,000 is low in comparison to 22.2 in Wigan. Liverpool and Middlesbrough hold the worst record after this.

The Goldsmiths' Company have offered, and the Senate of the University of London has accepted, the site and building of the Goldsmiths' Institute at New Cross. This gift is of an estimated value of not less than £100,000. The institute commenced its work in 1891, and has received within it 100,000 students.

**The late Mrs. M. Chrimes, of Rotherham, has bequeathed £1,000 each to the Rotherham Hospital, the Jessop Hospital for Women, Sheffield, the Devonshire Hospital, Buxton, the Scarborough Hospital, and the Sheffield Infirmary. The late Mr. H. Faulenbach, of Enfield, has bequeathed £500 to the German Hospital.**

The Paris Faculty of Medicine has opened the new laboratory of experimental medicine at Port de Vanne. The laboratory is for experiments on animals, and is under the direction of the Faculty, which paid part of the cost of the buildings and equipment, the city presenting the site, and private donors providing other necessary adjuncts.

A Lady residing at Hampstead having written to the Princess of Wales, calling attention to the danger arising from the presence on Hampstead Heath of consumption patients from the Hampstead Infirmary, and Mount Vernon Hospital, and alleging that these people were not provided with sputa flasks, and thus constituted a public nuisance, her Royal Highness forwarded the letter to the Local Government Board. On Friday last the matter was, in consequence, discussed by the Hampstead Guardians, one of the Local Government Board Inspectors being present. It having been stated that whenever the patients of either the Infirmary or hospital went out they carried flasks, the Inspector expressed himself as satisfied that there was no cause for complaint in respect to them.

